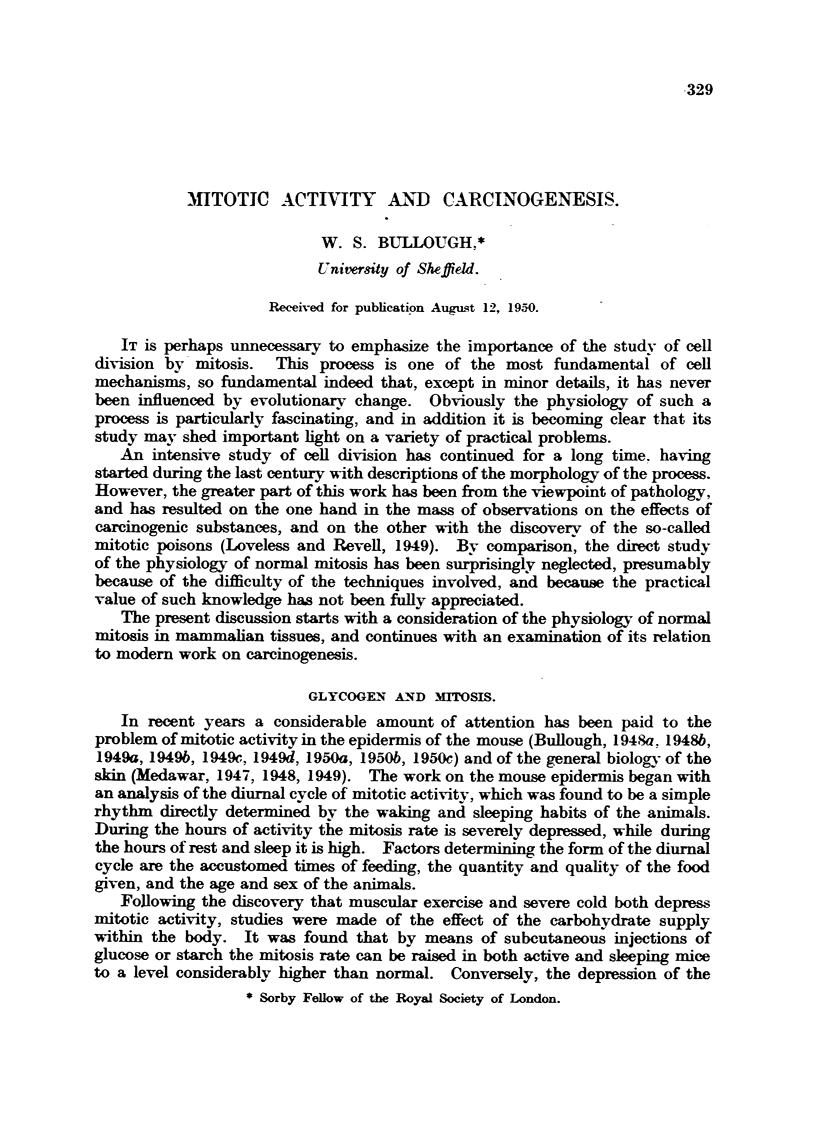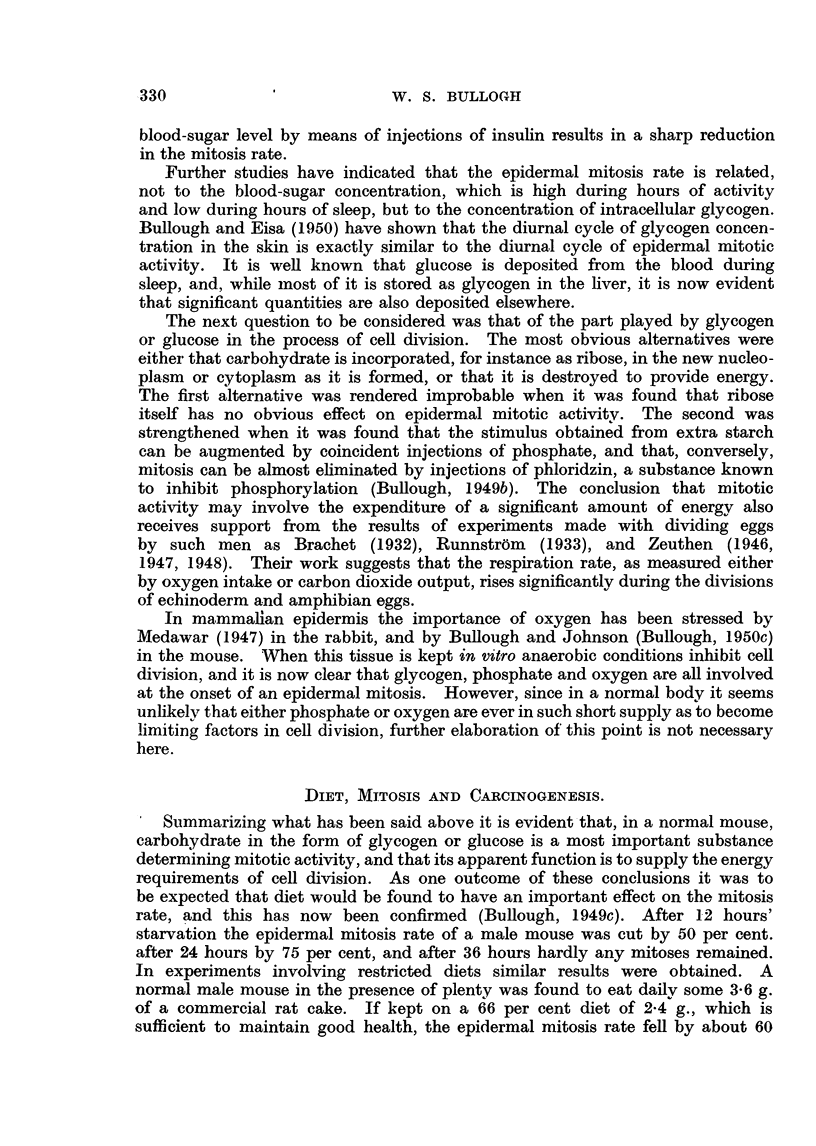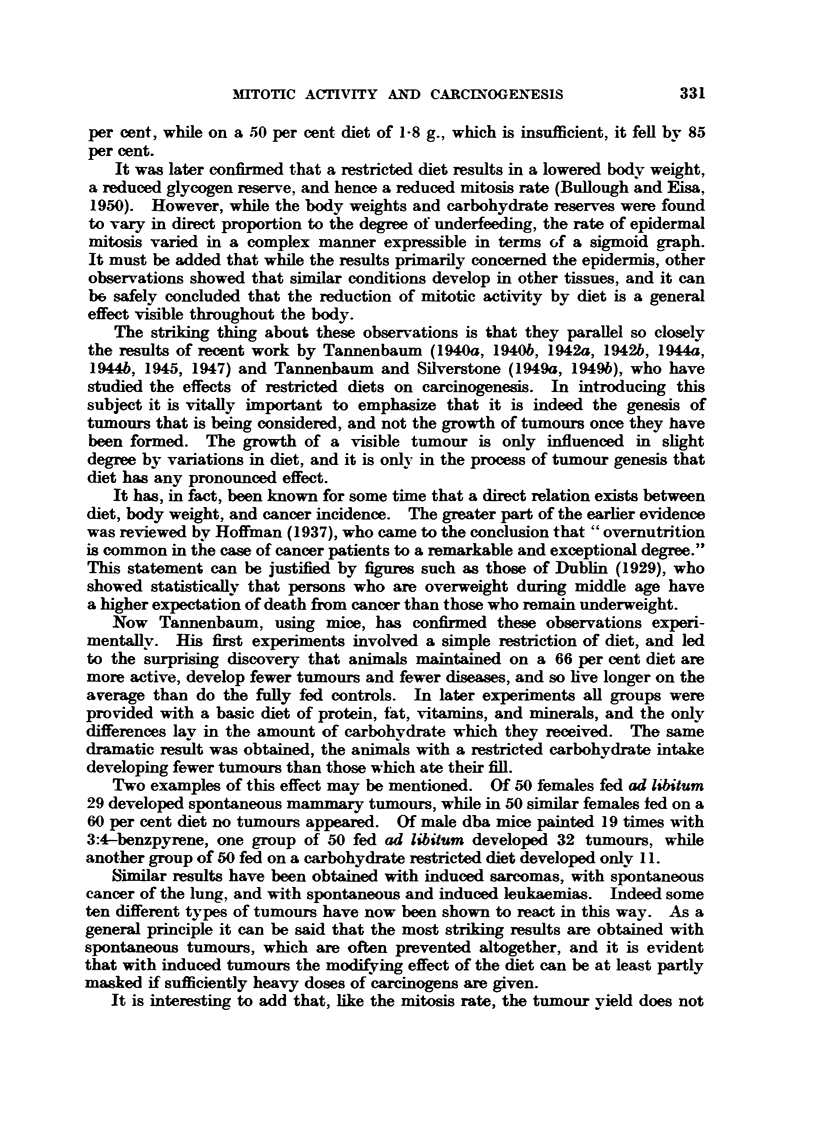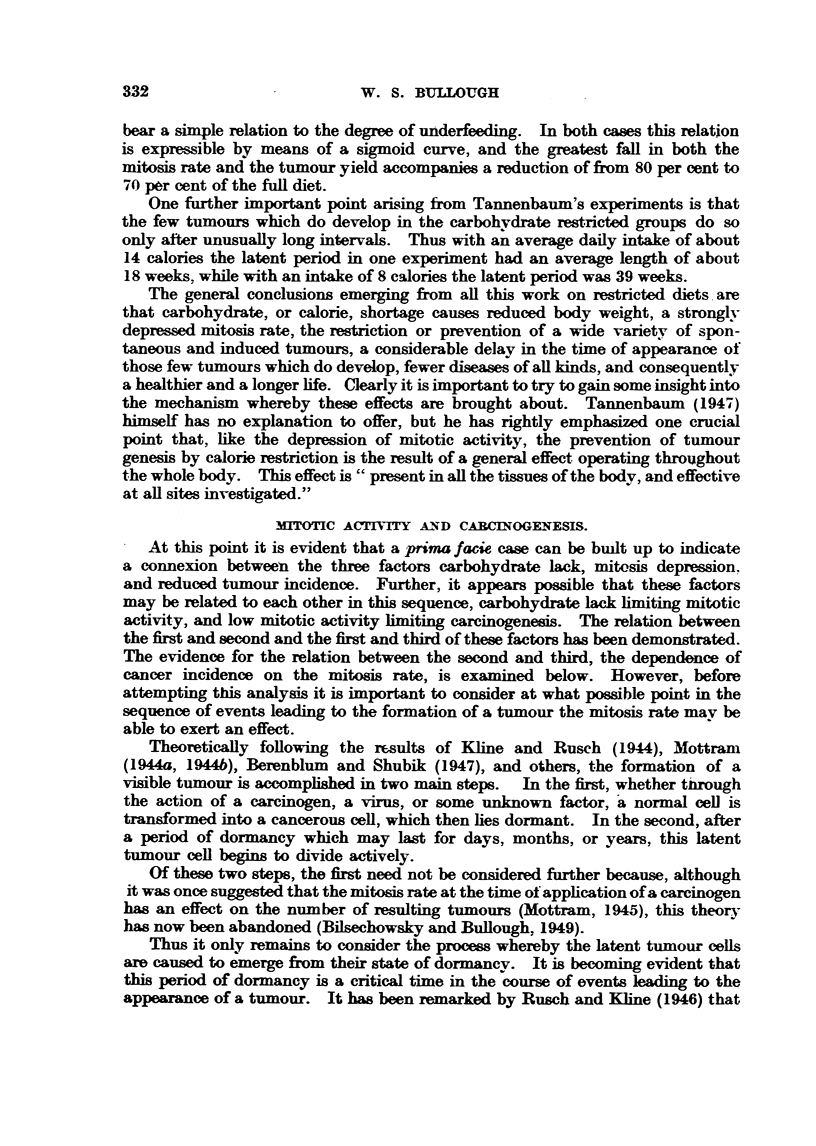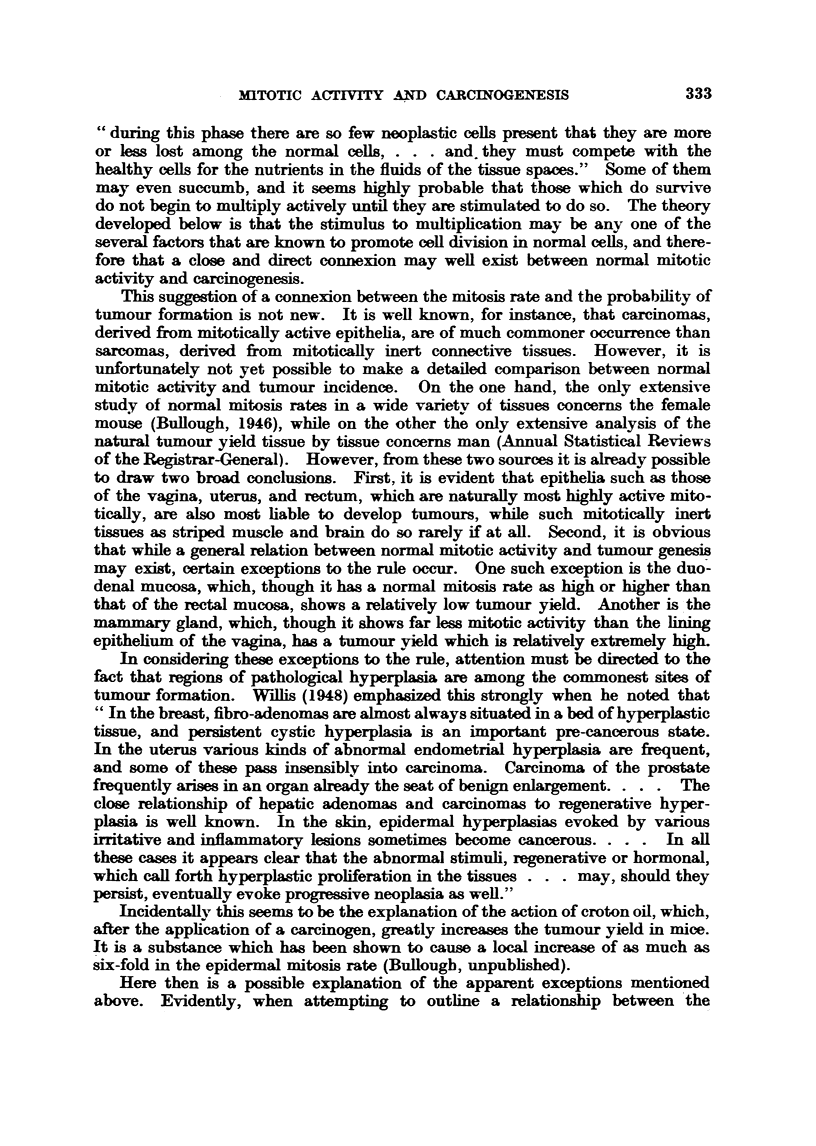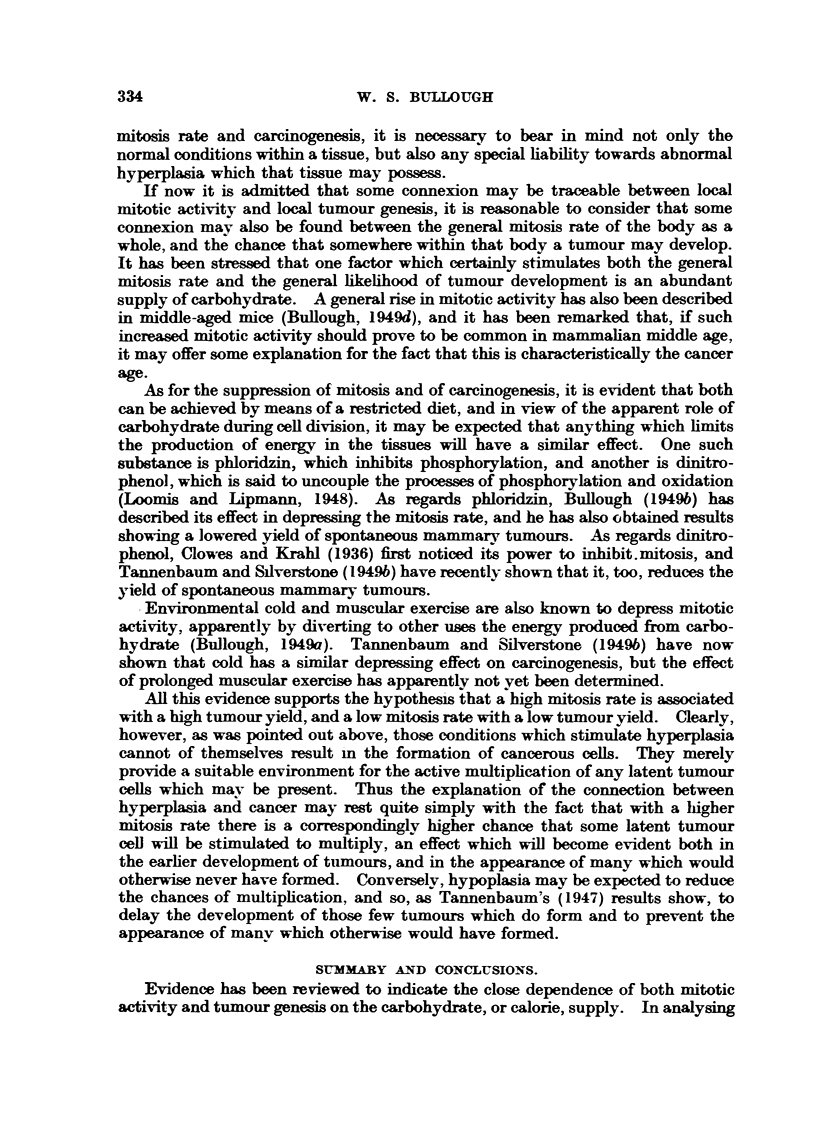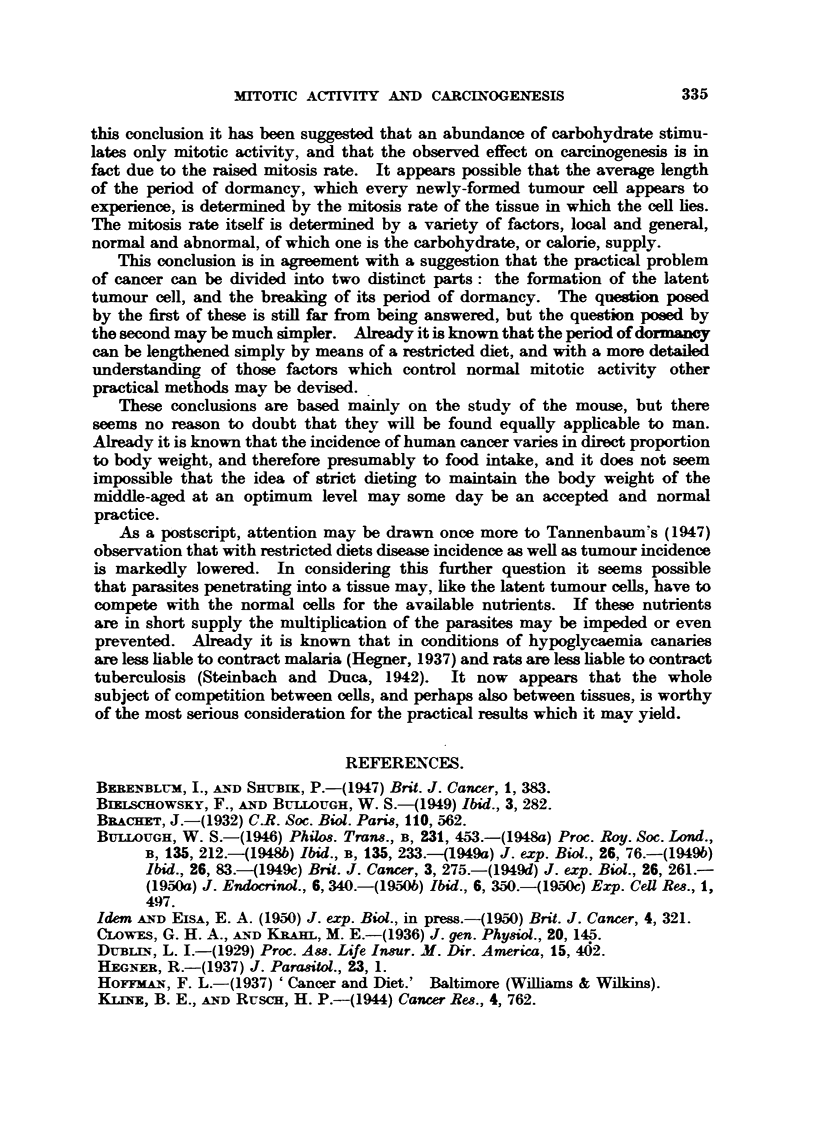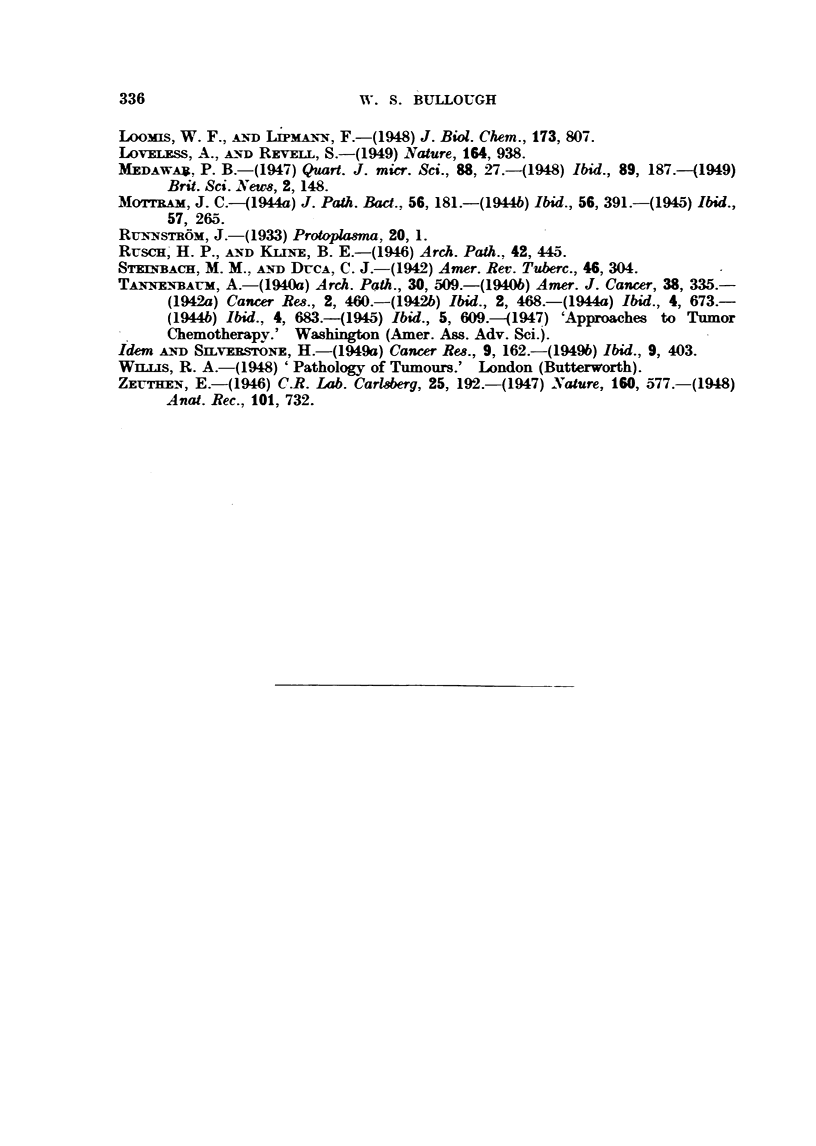# Mitotic Activity and Carcinogenesis

**DOI:** 10.1038/bjc.1950.31

**Published:** 1950-09

**Authors:** W. S. Bullough


### MITOTIC ACTIVITY AND CARCINOGENESIS.

W. S. BULLOUGH,*

*University of Sheffield.*

Received for publication August 12, 1950.

It is perhaps unnecessary to emphasize the importance of the study of cell division by mitosis. This process is one of the most fundamental of cell mechanisms, so fundamental indeed that, except in minor details, it has never been influenced by evolutionary change. Obviously the physiology of such a process is particularly fascinating, and in addition it is becoming clear that its study may shed important light on a variety of practical problems.

An intensive study of cell division has continued for a long time, having started during the last century with descriptions of the morphology of the process. However, the greater part of this work has been from the viewpoint of pathology, and has resulted on the one hand in the mass of observations on the effects of carcinogenic substances, and on the other with the discovery of the so-called mitotic poisons (Loveless and Revell, 1949). By comparison, the direct study of the physiology of normal mitosis has been surprisingly neglected, presumably because of the difficulty of the techniques involved, and because the practical value of such knowledge has not been fully appreciated.

The present discussion starts with a consideration of the physiology of normal mitosis in mammalian tissues, and continues with an examination of its relation to modern work on carcinogenesis.

### GLYCOGEN AND MITOSIS.

In recent years a considerable amount of attention has been paid to the problem of mitotic activity in the epidermis of the mouse (Bullough, 1948*a*, 1948*b*, 1949*a*, 1949*b*, 1949*c*, 1949*d*, 1950*a*, 1950*b*, 1950*c*) and of the general biology of the skin (Medawar, 1947, 1948, 1949). The work on the mouse epidermis began with an analysis of the diurnal cycle of mitotic activity, which was found to be a simple rhythm directly determined by the waking and sleeping habits of the animals. During the hours of activity the mitosis rate is severely depressed, while during the hours of rest and sleep it is high. Factors determining the form of the diurnal cycle are the accustomed times of feeding, the quantity and quality of the food given, and the age and sex of the animals.

Following the discovery that muscular exercise and severe cold both depress mitotic activity, studies were made of the effect of the carbohydrate supply within the body. It was found that by means of subcutaneous injections of glucose or starch the mitosis rate can be raised in both active and sleeping mice to a level considerably higher than normal. Conversely, the depression of the

* Sorby Fellow of the Royal Society of London.

blood-sugar level by means of injections of insulin results in a sharp reduction in the mitosis rate.

Further studies have indicated that the epidermal mitosis rate is related, not to the blood-sugar concentration, which is high during hours of activity and low during hours of sleep, but to the concentration of intracellular glycogen. Bullough and Eisa (1950) have shown that the diurnal cycle of glycogen concentration in the skin is exactly similar to the diurnal cycle of epidermal mitotic activity. It is well known that glucose is deposited from the blood during sleep, and, while most of it is stored as glycogen in the liver, it is now evident that significant quantities are also deposited elsewhere.

The next question to be considered was that of the part played by glycogen or glucose in the process of cell division. The most obvious alternatives were either that carbohydrate is incorporated, for instance as ribose, in the new nucleoplasm or cytoplasm as it is formed, or that it is destroyed to provide energy. The first alternative was rendered improbable when it was found that ribose itself has no obvious effect on epidermal mitotic activity. The second was strengthened when it was found that the stimulus obtained from extra starch can be augmented by coincident injections of phosphate, and that, conversely, mitosis can be almost eliminated by injections of phloridzin, a substance known to inhibit phosphorylation (Bullough, 1949*b*). The conclusion that mitotic activity may involve the expenditure of a significant amount of energy also receives support from the results of experiments made with dividing eggs by such men as Brachet (1932), Runnström (1933), and Zeuthen (1946, 1947, 1948). Their work suggests that the respiration rate, as measured either by oxygen intake or carbon dioxide output, rises significantly during the divisions of echinoderm and amphibian eggs.

In mammalian epidermis the importance of oxygen has been stressed by Medawar (1947) in the rabbit, and by Bullough and Johnson (Bullough, 1950*c*) in the mouse. When this tissue is kept *in vitro* anaerobic conditions inhibit cell division, and it is now clear that glycogen, phosphate and oxygen are all involved at the onset of an epidermal mitosis. However, since in a normal body it seems unlikely that either phosphate or oxygen are ever in such short supply as to become limiting factors in cell division, further elaboration of this point is not necessary here.

### Diet, Mitosis and Carcinogenesis.

Summarizing what has been said above it is evident that, in a normal mouse, carbohydrate in the form of glycogen or glucose is a most important substance determining mitotic activity, and that its apparent function is to supply the energy requirements of cell division. As one outcome of these conclusions it was to be expected that diet would be found to have an important effect on the mitosis rate, and this has now been confirmed (Bullough, 1949*c*). After 12 hours' starvation the epidermal mitosis rate of a male mouse was cut by 50 per cent. after 24 hours by 75 per cent, and after 36 hours hardly any mitoses remained. In experiments involving restricted diets similar results were obtained. A normal male mouse in the presence of plenty was found to eat daily some 3·6 g. of a commercial rat cake. If kept on a 66 per cent diet of 2·4 g., which is sufficient to maintain good health, the epidermal mitosis rate fell by about 60

per cent, while on a 50 per cent diet of 1·8 g., which is insufficient, it fell by 85 per cent.

It was later confirmed that a restricted diet results in a lowered body weight, a reduced glycogen reserve, and hence a reduced mitosis rate (Bullough and Eisa, 1950). However, while the body weights and carbohydrate reserves were found to vary in direct proportion to the degree of underfeeding, the rate of epidermal mitosis varied in a complex manner expressible in terms of a sigmoid graph. It must be added that while the results primarily concerned the epidermis, other observations showed that similar conditions develop in other tissues, and it can be safely concluded that the reduction of mitotic activity by diet is a general effect visible throughout the body.

The striking thing about these observations is that they parallel so closely the results of recent work by Tannenbaum (1940*a*, 1940*b*, 1942*a*, 1942*b*, 1944*a*, 1944*b*, 1945, 1947) and Tannenbaum and Silverstone (1949*a*, 1949*b*), who have studied the effects of restricted diets on carcinogenesis. In introducing this subject it is vitally important to emphasize that it is indeed the genesis of tumours that is being considered, and not the growth of tumours once they have been formed. The growth of a visible tumour is only influenced in slight degree by variations in diet, and it is only in the process of tumour genesis that diet has any pronounced effect.

It has, in fact, been known for some time that a direct relation exists between diet, body weight, and cancer incidence. The greater part of the earlier evidence was reviewed by Hoffman (1937), who came to the conclusion that "overnutrition is common in the case of cancer patients to a remarkable and exceptional degree." This statement can be justified by figures such as those of Dublin (1929), who showed statistically that persons who are overweight during middle age have a higher expectation of death from cancer than those who remain underweight.

Now Tannenbaum, using mice, has confirmed these observations experimentally. His first experiments involved a simple restriction of diet, and led to the surprising discovery that animals maintained on a 66 per cent diet are more active, develop fewer tumours and fewer diseases, and so live longer on the average than do the fully fed controls. In later experiments all groups were provided with a basic diet of protein, fat, vitamins, and minerals, and the only differences lay in the amount of carbohydrate which they received. The same dramatic result was obtained, the animals with a restricted carbohydrate intake developing fewer tumours than those which ate their fill.

Two examples of this effect may be mentioned. Of 50 females fed *ad libitum* 29 developed spontaneous mammary tumours, while in 50 similar females fed on a 60 per cent diet no tumours appeared. Of male dba mice painted 19 times with 3:4–benzpyrene, one group of 50 fed *ad libitum* developed 32 tumours, while another group of 50 fed on a carbohydrate restricted diet developed only 11.

Similar results have been obtained with induced sarcomas, with spontaneous cancer of the lung, and with spontaneous and induced leukaemias. Indeed some ten different types of tumours have now been shown to react in this way. As a general principle it can be said that the most striking results are obtained with spontaneous tumours, which are often prevented altogether, and it is evident that with induced tumours the modifying effect of the diet can be at least partly masked if sufficiently heavy doses of carcinogens are given.

It is interesting to add that, like the mitosis rate, the tumour yield does not bear a simple relation to the degree of underfeeding. In both cases this relation is expressible by means of a sigmoid curve, and the greatest fall in both the mitosis rate and the tumour yield accompanies a reduction of from 80 per cent to 70 per cent of the full diet.

One further important point arising from Tannenbaum's experiments is that the few tumours which do develop in the carbohydrate restricted groups do so only after unusually long intervals. Thus with an average daily intake of about 14 calories the latent period in one experiment had an average length of about 18 weeks, while with an intake of 8 calories the latent period was 39 weeks.

The general conclusions emerging from all this work on restricted diets are that carbohydrate, or calorie, shortage causes reduced body weight, a strongly depressed mitosis rate, the restriction or prevention of a wide variety of spontaneous and induced tumours, a considerable delay in the time of appearance of those few tumours which do develop, fewer diseases of all kinds, and consequently a healthier and a longer life. Clearly it is important to try to gain some insight into the mechanism whereby these effects are brought about. Tannenbaum (1947) himself has no explanation to offer, but he has rightly emphasized one crucial point that, like the depression of mitotic activity, the prevention of tumour genesis by calorie restriction is the result of a general effect operating throughout the whole body. This effect is " present in all the tissues of the body, and effective at all sites investigated."

### MITOTIC ACTIVITY AND CARCINOGENESIS.

At this point it is evident that a *prima facie* case can be built up to indicate a connexion between the three factors carbohydrate lack, mitosis depression, and reduced tumour incidence. Further, it appears possible that these factors may be related to each other in this sequence, carbohydrate lack limiting mitotic activity, and low mitotic activity limiting carcinogenesis. The relation between the first and second and the first and third of these factors has been demonstrated. The evidence for the relation between the second and third, the dependence of cancer incidence on the mitosis rate, is examined below. However, before attempting this analysis it is important to consider at what possible point in the sequence of events leading to the formation of a tumour the mitosis rate may be able to exert an effect.

Theoretically following the results of Kline and Rusch (1944), Mottram (1944*a*, 1944*b*), Berenblum and Shubik (1947), and others, the formation of a visible tumour is accomplished in two main steps. In the first, whether through the action of a carcinogen, a virus, or some unknown factor, a normal cell is transformed into a cancerous cell, which then lies dormant. In the second, after a period of dormancy which may last for days, months, or years, this latent tumour cell begins to divide actively.

Of these two steps, the first need not be considered further because, although it was once suggested that the mitosis rate at the time of application of a carcinogen has an effect on the number of resulting tumours (Mottram, 1945), this theory has now been abandoned (Bilsechowsky and Bullough, 1949).

Thus it only remains to consider the process whereby the latent tumour cells are caused to emerge from their state of dormancy. It is becoming evident that this period of dormancy is a critical time in the course of events leading to the appearance of a tumour. It has been remarked by Rusch and Kline (1946) that

" during this phase there are so few neoplastic cells present that they are more or less lost among the normal cells, . . . and they must compete with the healthy cells for the nutrients in the fluids of the tissue spaces." Some of them may even succumb, and it seems highly probable that those which do survive do not begin to multiply actively until they are stimulated to do so. The theory developed below is that the stimulus to multiplication may be any one of the several factors that are known to promote cell division in normal cells, and therefore that a close and direct connexion may well exist between normal mitotic activity and carcinogenesis.

This suggestion of a connexion between the mitosis rate and the probability of tumour formation is not new. It is well known, for instance, that carcinomas, derived from mitotically active epithelia, are of much commoner occurrence than sarcomas, derived from mitotically inert connective tissues. However, it is unfortunately not yet possible to make a detailed comparison between normal mitotic activity and tumour incidence. On the one hand, the only extensive study of normal mitosis rates in a wide variety of tissues concerns the female mouse (Bullough, 1946), while on the other the only extensive analysis of the natural tumour yield tissue by tissue concerns man (Annual Statistical Reviews of the Registrar-General). However, from these two sources it is already possible to draw two broad conclusions. First, it is evident that epithelia such as those of the vagina, uterus, and rectum, which are naturally most highly active mitotically, are also most liable to develop tumours, while such mitotically inert tissues as striped muscle and brain do so rarely if at all. Second, it is obvious that while a general relation between normal mitotic activity and tumour genesis may exist, certain exceptions to the rule occur. One such exception is the duodenal mucosa, which, though it has a normal mitosis rate as high or higher than that of the rectal mucosa, shows a relatively low tumour yield. Another is the mammary gland, which, though it shows far less mitotic activity than the lining epithelium of the vagina, has a tumour yield which is relatively extremely high.

In considering these exceptions to the rule, attention must be directed to the fact that regions of pathological hyperplasia are among the commonest sites of tumour formation. Willis (1948) emphasized this strongly when he noted that " In the breast, fibro-adenomas are almost always situated in a bed of hyperplastic tissue, and persistent cystic hyperplasia is an important pre-cancerous state. In the uterus various kinds of abnormal endometrial hyperplasia are frequent, and some of these pass insensibly into carcinoma. Carcinoma of the prostate frequently arises in an organ already the seat of benign enlargement. . . . The close relationship of hepatic adenomas and carcinomas to regenerative hyperplasia is well known. In the skin, epidermal hyperplasias evoked by various irritative and inflammatory lesions sometimes become cancerous. . . . In all these cases it appears clear that the abnormal stimuli, regenerative or hormonal, which call forth hyperplastic proliferation in the tissues . . . may, should they persist, eventually evoke progressive neoplasia as well."

Incidentally this seems to be the explanation of the action of croton oil, which, after the application of a carcinogen, greatly increases the tumour yield in mice. It is a substance which has been shown to cause a local increase of as much as six-fold in the epidermal mitosis rate (Bullough, unpublished).

Here then is a possible explanation of the apparent exceptions mentioned above. Evidently, when attempting to outline a relationship between the mitosis rate and carcinogenesis, it is necessary to bear in mind not only the normal conditions within a tissue, but also any special liability towards abnormal hyperplasia which that tissue may possess.

If now it is admitted that some connexion may be traceable between local mitotic activity and local tumour genesis, it is reasonable to consider that some connexion may also be found between the general mitosis rate of the body as a whole, and the chance that somewhere within that body a tumour may develop. It has been stressed that one factor which certainly stimulates both the general mitosis rate and the general likelihood of tumour development is an abundant supply of carbohydrate. A general rise in mitotic activity has also been described in middle-aged mice (Bullough, 1949*d*), and it has been remarked that, if such increased mitotic activity should prove to be common in mammalian middle age, it may offer some explanation for the fact that this is characteristically the cancer age.

As for the suppression of mitosis and of carcinogenesis, it is evident that both can be achieved by means of a restricted diet, and in view of the apparent role of carbohydrate during cell division, it may be expected that anything which limits the production of energy in the tissues will have a similar effect. One such substance is phloridzin, which inhibits phosphorylation, and another is dinitrophenol, which is said to uncouple the processes of phosphorylation and oxidation (Loomis and Lipmann, 1948). As regards phloridzin, Bullough (1949*b*) has described its effect in depressing the mitosis rate, and he has also obtained results showing a lowered yield of spontaneous mammary tumours. As regards dinitrophenol, Clowes and Krahl (1936) first noticed its power to inhibit.mitosis, and Tannenbaum and Silverstone (1949*b*) have recently shown that it, too, reduces the yield of spontaneous mammary tumours.

Environmental cold and muscular exercise are also known to depress mitotic activity, apparently by diverting to other uses the energy produced from carbohydrate (Bullough, 1949*a*). Tannenbaum and Silverstone (1949*b*) have now shown that cold has a similar depressing effect on carcinogenesis, but the effect of prolonged muscular exercise has apparently not yet been determined.

All this evidence supports the hypothesis that a high mitosis rate is associated with a high tumour yield, and a low mitosis rate with a low tumour yield. Clearly, however, as was pointed out above, those conditions which stimulate hyperplasia cannot of themselves result in the formation of cancerous cells. They merely provide a suitable environment for the active multiplication of any latent tumour cells which may be present. Thus the explanation of the connection between hyperplasia and cancer may rest quite simply with the fact that with a higher mitosis rate there is a correspondingly higher chance that some latent tumour cell will be stimulated to multiply, an effect which will become evident both in the earlier development of tumours, and in the appearance of many which would otherwise never have formed. Conversely, hypoplasia may be expected to reduce the chances of multiplication, and so, as Tannenbaum's (1947) results show, to delay the development of those few tumours which do form and to prevent the appearance of many which otherwise would have formed.

### SUMMARY AND CONCLUSIONS.

Evidence has been reviewed to indicate the close dependence of both mitotic activity and tumour genesis on the carbohydrate, or calorie, supply. In analysing this conclusion it has been suggested that an abundance of carbohydrate stimulates only mitotic activity, and that the observed effect on carcinogenesis is in fact due to the raised mitosis rate. It appears possible that the average length of the period of dormancy, which every newly-formed tumour cell appears to experience, is determined by the mitosis rate of the tissue in which the cell lies. The mitosis rate itself is determined by a variety of factors, local and general, normal and abnormal, of which one is the carbohydrate, or calorie, supply.

This conclusion is in agreement with a suggestion that the practical problem of cancer can be divided into two distinct parts : the formation of the latent tumour cell, and the breaking of its period of dormancy. The question posed by the first of these is still far from being answered, but the question posed by the second may be much simpler. Already it is known that the period of dormancy can be lengthened simply by means of a restricted diet, and with a more detailed understanding of those factors which control normal mitotic activity other practical methods may be devised.

These conclusions are based mainly on the study of the mouse, but there seems no reason to doubt that they will be found equally applicable to man. Already it is known that the incidence of human cancer varies in direct proportion to body weight, and therefore presumably to food intake, and it does not seem impossible that the idea of strict dieting to maintain the body weight of the middle-aged at an optimum level may some day be an accepted and normal practice.

As a postscript, attention may be drawn once more to Tannenbaum's (1947) observation that with restricted diets disease incidence as well as tumour incidence is markedly lowered. In considering this further question it seems possible that parasites penetrating into a tissue may, like the latent tumour cells, have to compete with the normal cells for the available nutrients. If these nutrients are in short supply the multiplication of the parasites may be impeded or even prevented. Already it is known that in conditions of hypoglycaemia canaries are less liable to contract malaria (Hegner, 1937) and rats are less liable to contract tuberculosis (Steinbach and Duca, 1942). It now appears that the whole subject of competition between cells, and perhaps also between tissues, is worthy of the most serious consideration for the practical results which it may yield.

### REFERENCES.

BERENBLUM, I., AND SHUBIK, P.—(1947) *Brit. J. Cancer*, **1**, 383.

BIELSCHOWSKY, F., AND BULLOUGH, W. S.—(1949) *Ibid.*, **3**, 282.

BRACHET, J.—(1932) *C.R. Soc. Biol. Paris*, **110**, 562.

BULLOUGH, W. S.—(1946) *Philos. Trans.*, B, **231**, 453.—(1948*a*) *Proc. Roy. Soc. Lond.*, B, **135**, 212.—(1948*b*) *Ibid.*, B, **135**, 233.—(1949*a*) *J. exp. Biol.*, **26**, 76.—(1949*b*) *Ibid.*, **26**, 83.—(1949*c*) *Brit. J. Cancer*, **3**, 275.—(1949*d*) *J. exp. Biol.*, **26**, 261.—(1950*a*) *J. Endocrinol.*, **6**, 340.—(1950*b*) *Ibid.*, **6**, 350.—(1950*c*) *Exp. Cell Res.*, **1**, 497.

*Idem* AND EISA, E. A. (1950) *J. exp. Biol.*, in press.—(1950) *Brit. J. Cancer*, **4**, 321.

CLOWES, G. H. A., AND KRAHL, M. E.—(1936) *J. gen. Physiol.*, **20**, 145.

DUBLIN, L. I.—(1929) *Proc. Ass. Life Insur. M. Dir. America*, **15**, 402.

HEGNER, R.—(1937) *J. Parasitol.*, **23**, 1.

HOFFMAN, F. L.—(1937) ' Cancer and Diet.' Baltimore (Williams & Wilkins).

KLINE, B. E., AND RUSCH, H. P.—(1944) *Cancer Res.*, **4**, 762.

LOOMIS, W. F., AND LIPMANN, F.—(1948) *J. Biol. Chem.*, **173**, 807.

LOVELESS, A., AND REVELL, S.—(1949) *Nature*, **164**, 938.

MEDAWAR, P. B.—(1947) *Quart. J. micr. Sci.*, **88**, 27.—(1948) *Ibid.*, **89**, 187.—(1949) *Brit. Sci. News*, **2**, 148.

MOTTRAM, J. C.—(1944*a*) *J. Path. Bact.*, **56**, 181.—(1944*b*) *Ibid.*, **56**, 391.—(1945) *Ibid.*, **57**, 265.

RUNNSTRÖM, J.—(1933) *Protoplasma*, **20**, 1.

RUSCH, H. P., AND KLINE, B. E.—(1946) *Arch. Path.*, **42**, 445.

STEINBACH, M. M., AND DUCA, C. J.—(1942) *Amer. Rev. Tuberc.*, **46**, 304.

TANNENBAUM, A.—(1940*a*) *Arch. Path.*, **30**, 509.—(1940*b*) *Amer. J. Cancer*, **38**, 335.—(1942*a*) *Cancer Res.*, **2**, 460.—(1942*b*) *Ibid.*, **2**, 468.—(1944*a*) *Ibid.*, **4**, 673.—(1944*b*) *Ibid.*, **4**, 683.—(1945) *Ibid.*, **5**, 609.—(1947) 'Approaches to Tumor Chemotherapy.' Washington (Amer. Ass. Adv. Sci.).

*Idem* AND SILVERSTONE, H.—(1949*a*) *Cancer Res.*, **9**, 162.—(1949*b*) *Ibid.*, **9**, 403.

WILLIS, R. A.—(1948) ' Pathology of Tumours.' London (Butterworth).

ZEUTHEN, E.—(1946) *C.R. Lab. Carlsberg*, **25**, 192.—(1947) *Nature*, **160**, 577.—(1948) *Anat. Rec.*, **101**, 732.